# Molecular Mechanism of *MYL4* Regulation of Skeletal Muscle Development in Pigs

**DOI:** 10.3390/genes14061267

**Published:** 2023-06-15

**Authors:** Xueli Xu, Zonggang Yu, Nini Ai, Sui Liufu, Xiaolin Liu, Bohe Chen, Xintong Li, Jun Jiang, Yuebo Zhang, Haiming Ma, Yulong Yin

**Affiliations:** 1 College of Animal Science and Technology, Hunan Agricultural University, Changsha 410128, China; xxl1632501152@163.com (X.X.); study236@163.com (Z.Y.); 15291270712@163.com (N.A.); liufusui0816@163.com (S.L.); lxl810711@163.com (X.L.); chenhe0213914@163.com (B.C.); p13548614212@163.com (X.L.); jiangjun1121@126.com (J.J.); ybzhangfd@126.com (Y.Z.); 2 Guangdong Laboratory for Lingnan Modern Agriculture, Guangzhou 510642, China; 3 Institute of Subtropical Agriculture, Chinese Academy of Sciences, Changsha 410125, China

**Keywords:** *MYL4*, Ningxiang pig, myoblast growth and development, C2C12

## Abstract

The processes of muscle growth and development, including myoblast proliferation, migration, differentiation, and fusion, are modified by a variety of regulatory factors. *MYL4* plays an important role in atrial development, atrial cardiomyopathy, muscle-fiber size, and muscle development. The structural variation (SV) of *MYL4* was found via the de novo sequencing of Ningxiang pigs, and the existence of SV was verified in the experiments. The genotype distribution of Ningxiang pigs and Large White pigs was detected, and it was found that Ningxiang pigs were mainly of the *BB* genotype and that Large White pigs were mainly of the *AB* genotype. However, the molecular mechanisms behind the *MYL4*-mediated regulation of skeletal muscle development need to be deeply explored. Therefore, RT-qPCR, 3′RACE, CCK8, EdU, Western blot, immunofluorescence, flow cytometry, and bioinformation analysis were used to explore the function of *MYL4* in myoblast development. The cDNA of *MYL4* was successfully cloned from Ningxiang pigs, and its physicochemical properties were predicted. The expression profiles in six tissues and four stages of Ningxiang pigs and Large White pigs were found to be the highest in the lungs and 30 days after birth. The expression of *MYL4* increased gradually with the extension of the myogenic differentiation time. The myoblast function test showed that the overexpression of *MYL4* inhibited proliferation and promoted apoptosis and differentiation. The knockdown of *MYL4* showed the opposite result. These results enhance our understanding of the molecular mechanisms of muscle development and provide a solid theoretical foundation for further exploring the role of the *MYL4* gene in muscle development.

## 1. Introduction

Due to rapid economic development and improved living standards, people have higher requirements in terms of the quality of livestock and poultry meat, and the demand for high-quality meat is increasing. Skeletal muscle is not only an important tissue in animals but also a source of daily meat products. Skeletal muscle consists of muscle fibers and connective tissue [1]. Muscle growth and development is closely related to meat production and is the main factor affecting overall growth. The maturation of muscle fibers goes through four stages: premyogenic progenitors, myoblasts, muscle tubes, and muscle fibers [2]. This complex process is precisely regulated by specific myogenic regulatory factors, such as the paired box family, the myocyte enhancer factor family, growth factors, cytokines, and other factors [3,4]. Genomic structural mutations are an important source of variation in many species and play an important role in phenotypic diversification and evolution [5]. Structural variation (SV) is defined as changes larger than 50 bp in the structure of a chromosome, and the main forms are deletion, insertion, repetition, inversion, and translocation [6]. Deletion is the most common type of SV, in which a nucleotide sequence is deleted on the chromosome, resulting in a decrease in the number of bases. Ma et al. identified 47 Chinese domestic pig-specific SVs; a 281 bp deletion in the first intron of the *MYL4* gene was found in these SVs, and the existence of the SV of *MYL4* was verified via experiments [7].

*MYL4*, also known as MLC1, encodes a kind of basic myosin light chain and is a member of the MYL family. The MYL family includes *MYL2*, *MYL1*, *MYL3*, and *MYL4*, and there is a high degree of sequence conservation among these genes. The *MYL4* gene was found to be located on chromosome 12 in pigs and on chromosome 11 in mice [8,9]. Studies have shown that *MYL4* was expressed in both adult atrial muscles and fetal skeletal muscles; two alternative splicing transcripts were found in the *MYL4* gene, encoding the MLC1_A_ subtype in adult atrial muscles and the MLCl_emb_ subtype in fetal skeletal muscles and showing the same protein-coding sequence [10]. At present, reports on *MYL4* are mostly focused on atrial development and atrial cardiomyopathy [11,12,13,14]. Moreover, *MYL4* can also play a regulatory role in the process of muscle development. *MYL4* constitutes the structural component of muscles and can regulate the development of muscle fibers, and it is related to muscle development and striated muscle contraction [15,16]. Ghazizadeh et al. found increased retinoic acid synthesis and actin disorder in *MYL4* mutant cell lines and zebrafish mutation models, indicating that *MYL4* interacts with cytoskeletal actin both in vitro and in vivo [17]. Dong et al. identified nine SNPs in Pig’s 5′ flanking region of *MYL4* genes; these nine SNPs increased the mRNA and protein expression of *MYL4* in porcine muscles and leads to an increase in the number of muscle fibers in porcine *Longissimus dorsi* muscle [18]. Given that *MYL4* is involved in the whole process of skeletal muscle, the effect of *MYL4* on cells within muscle tissue requires further exploration.

In this experiment, we detected the distribution SV in *MYL4* in Ningxiang pigs and Large White pigs and cloned the sequence of *MYL4* in Ningxiang pigs and predicted its physicochemical properties. We also analyzed the tissue expression profiles of *MYL4* and determined its role in C2C12 cells. Our results showed that *MYL4* inhibits the proliferation of C2C12 cells and promotes the differentiation and apoptosis of C2C12 cells.

## 2. Materials and Methods

### 2.1. Animals and Samples

The animals used in the experiments were Ningxiang pigs and Large White pigs. Ningxiang pigs were purchased from Hunan Ningxiang Dalong Animal Husbandry Technology Co., Ltd. (Changsha, China). The Large White pigs were purchased from Xiangtan Weihong Food Co., Ltd. (Changsha, China). The ears of the Ningxiang pigs (n = 110) and the Large White pigs (n = 110) were collected and stored at −80 °C. Samples of the hearts, livers, spleens, lungs, fat, and *Longissimus dorsi* muscles of 30, 90, 150, and 210 days-after-birth Ningxiang pigs (n = 3) and Large White pigs (n = 3) were collected and stored in liquid nitrogen immediately. The *Longissimus dorsi* muscle of a 1-day-old Ningxiang pig was collected to clone the cDNA sequence of *MYL4*. All of the studies involving animals were conducted according to the ethics committee of the Animal Science and Technology College of Hunan Agriculture University (No. 2021–13).

### 2.2. Cell Culture and Transfection

The C2C12 cells were purchased from Anweisci (Shanghai, China). The cells were cultured in a complete medium containing 89% DMEM (Gibco, Waltham, MA, USA), 10% fetal bovine serum (Gibco, Waltham, MA, USA) and 1% penicillin–streptomycin (Gibco, Waltham, MA, USA) and cultured in a 37 °C incubator containing 5% CO_2_. For myogenic differentiation, the complete medium was changed into DMEM containing 2% horse serum (Hyclone, Logan, UT, USA). Three siRNAs, Si-NC, PCDNA3.1-*MYL4* and PCDNA3.1, were purchased from JTS Scientific (Wuhan, China); the sequences were as follows (Table 1). The C2C12 cells were seeded into 6-well plates after reaching 80% confluence, and Si-*MYL4*, Si-NC, PCDNA3.1-*MYL4* and PCDNA3.1 were transfected into the cells using Lipofectamine 2000 (Invitrogen, Waltham, MA, USA) after reaching 50% confluence.

### 2.3. PCR

The DNA was separated using the TIANamp genomic DNA Kit (TIANGEN, Beijing, China), following the recommendations of the manufacturer. NanoDrop 2000 (Thermo Scientific, USA) was used to determine the purity and concentration of the DNA. The sequences of the primer used to detect the SV in *MYL4* were 5′TACTAGCTGCCACCTTGG 3′ (ssc-*MYL4*-1-F) and 5′ACAATGCCAGATCCTTAGCC 3′ (ssc-*MYL4*-1-R). The sequences of the primer used to clone the sequence of CDS were 5′CGCGTCTCTTGAGTCCTTCC3′ (*MYL4*-F) and 5′GTCTGCTTCACCCAGACATGA3′ (*MYL4*-R). PCR was performed using the Taq Master Mix buffer (Vazyme, Nanjing, China) on a PCR instrument (Bio-Rad, Hercules, CA, USA), and then the PCR production was detected using agarose gel electrophoresis. DL2000 was used as a DNA marker (Biodragon, Suzhou, China).

### 2.4. Real-Time Quantitative PCR

The RNA was extracted using the RNAsimple Total RNA kit (TIANGEN, Beijing, China), and the concentration and purity of the RNA were determined using agarose gel electrophoresis and NanoDrop 2000 (Thermo Scientific, Waltham, MA, USA). RNA reverse transcription was performed using a reverse transcription kit (Thermo Scientific, Waltham, MA, USA). PerfectStart^®^ Green qPCR SuperMix (TransGen, Beijing, China) was used for real-time quantitative PCR (RT-qPCR), and RT-qPCR was performed on a CFX connect real-time system (Bio-Rad, Hercules, CA, USA) using *Gapdh* for the reference genes. The relative expression of the genes was calculated using 2^−ΔΔCt^. The sequences of the genes were downloaded from the NCBI database (https://www.ncbi.nlm.nih.gov) (accessed on 9 May 2023), and Primer 5.0 was used to design the primers. All of the primers were synthesized from Tsingke Biotechnology (Beijing, China), and the primer sequences are listed in the following table (Table 2).

### 2.5. Cloning of 3′ Sequence of MYL4 cDNA

We performed 3′ RACE using SMARTer RACE 5′/3′ Kit (Takara, Dalian, Chain), following the recommendations of the manufacturer. The sequence of the primer used for 3′ RACE was 5′-CCAAGCCAGAAGAAATGAATGCC-3′. The first-stand cDNA of Ningxiang pigs’ *Longissimus dorsi* was used to clone the 3′ sequence of *MYL4*. Then, the PCR reaction was performed, and the PCR product was gel-purified using the TIANgel purification kit (TIANGEN, Beijing, China). Next, the purified-PCR product was ligated into the pMD18-Tvector (Takara, Dalian, Chain) and then transformed into DH5α-competent cells. Positive clones were selected and sequenced by Tsingke Biotechnology (Beijing, China).

### 2.6. Cell Proliferation Assays

Cell proliferation was measured using CCK-8 assays and EdU staining. For CCK-8 assays, the transfected C2C12 cells were seeded into a 96-well plate, 10 μL of CCK-8 solution was added after incubation for 0 h (cell adhesion) 12 h, 24 h, 36 h and 48 h. Then, the cells were incubated for 4 h. Finally, a microplate reader (Multiskan FC, Thermo Scientific, Waltham, MA, USA) was used to measure the absorbance at 450 nm. For EdU staining, the cells were incubated with DMEM containing 20 μM EdU solution for 2 h after reaching 80% confluence. Next, EdU staining was performed using the EdU kit (Meilunbio, Dalian, China), following the recommendations of the manufacturer. Finally, fluorescence microscopy (Axio Vert A1, ZEISS, Germany) was used to capture the images.

### 2.7. Western Blot

The cells were collected, and 150 μL of RIPA lysis buffer (Beyotime, Shanghai, China) containing 1% protease inhibitor (Beyotime, Shanghai, China) was added to extract the protein. The concentration of protein was measured using a BCA protein assay kit (Meilunbio, Dalian, China) on a Multiskan FC microplate reader (Thermo Scientific, Waltham, MA, USA). Then, 2× protein loading buffer (Solarbio, Beijing, China) was added to the protein, and the protein was denatured by heating in 100 °C water for 10 min. PAGE Gel was made using a PAGE Gel Fast Preparation Kit (Epizyme, Shanghai, China). A total of 4 µg of protein in each well of PAGE Gel was electrophoresed and then transferred to a polyvinylidene difluoride (PVDF) membrane (Beyotime, Shanghai, China). The PVDF membrane was blocked in blocking buffer (Beyotime, Shanghai, China) for 2 h and then incubated with primary antibodies overnight at 4 °C. The primary antibodies used for Western blot were as follows: Anti-CDK4 (AF300822, 1:1000, AiFangBio, Changsha, China), Anti-PCNA (R25293, 1:1000, Zenbio, Chengdu, China), Anti-BAX (R22708, 1:1000, Zenbio, Chengdu, China), Anti-Caspase3 (R22811, 1:1000, Zenbio, Chengdu, China), Anti-β-actin (HC201, 1:10,000, TransGen, Beijing, China), Anti-MyoG (F5D, 1:200, DSHB, Iowa, IA, USA), Anti-MyHC (MF20, 1:500, DSHB, Iowa, IA, USA), Anti-MyoD (ER1913-45, 1:1000, Huabio, Hangzhou, China), and Anti-MYL4 (AF07872, 1:500, AiFangBio, Changsha, China). Next, The PVDF membrane was incubated with secondary antibodies (1:15,000, Zenbio, Chengdu, China) for 2 h. Finally, the PVDF membrane was exposed using an Image Quant LAS 4000 mini (GE, Boston, MA, USA). The ratio of the target protein to the reference protein β-actin is the relative expression of each protein.

### 2.8. Flow Cytometry

Flow cytometry was used to evaluate cell apoptosis. The cells after transfection with Si-*MYL4*, Si-NC, PCDNA3.1-*MYL4*, and PCDNA3.1 were collected, and then 1 mL of ice-cold PBS was added to wash the cells. Then, 100 μL of binding buffer was added to disperse the cells, and the collected cells were treated with 5 μL Annexin V-FITC and 10 μL propidium iodide (Yeasen, Shanghai, China) in the dark for 15 min at 37 °C. Finally, the treated cells were added to 400 μL 1 × Binding Buffer and measured using Cytek DxP Athena flow cytometry (Cytek, Fremont, CA, USA).

### 2.9. Immunofluorescence Analysis

C2C12 cell differentiation was evaluated via immunofluorescence analysis. The differentiated cells were fixed using 4% paraformaldehyde (Beyotime, Shanghai, China) for 30 min, 0.5% triton X-100 (Solarbio, Beijing, China) for 20 min, and blocked with 5% bovine serum albumin (BioFroxx, Frankfurt, Germany) for 2 h. For myogenic differentiation at 4 d after transfection with Si-*MYL4*, Si-NC, the cells were incubated with anti-MyHC monoclonal antibody (MF20, 1:300, DSHB, Iowa, IA, USA) overnight at 4 °C. For myogenic differentiation at 0 d, 2 d, 4 d, 6 d, and 8 d, the cells were incubated with anti-MYL4 polyclonal antibody (67533-1-Ig, 1:500, proteintech, Chicago, IL, USA) overnight at 4 °C. Then, the cells were incubated with DyLight 488 goat anti-mouse IgG (1:1000, Abbkine, Wuhan, China) for 2 h. Next, the nuclei were stained with DAPI (1:100, Beyotime, Shanghai, China) for 10 min. Finally, images were captured using a fluorescence microscope (Axio Vert A1, ZEISS, Oberkochen, Germany). The number of nucleus in myotubes to total nucleus was differentiation index. Myotubes were classified into three levels (<3 nucleus, 3–5 nucleus, >5 nucleus), and the fusion index was defined as the number of nucleus in MyHC-positive myotubes (<3 nucleus, 3–5 nucleus, >5 nucleus) to total nucleus within MyHC-positive myotubes.

### 2.10. Bioinformatics Analysis

An open reading frame (ORF) was found on an online website “https://www.ncbi.nlm.nih.gov/orffinder” (accessed on 8 May 2023). Sequence blast using DNAMAN (Version 9) and blastn suite was enabled through an online website “https://blast.ncbi.nlm.nih.gov/blast” (accessed on 8 May 2023). A phylogenetic tree was constructed with the MYL4 protein of different species using the neighbor-joining (NJ) method and MEGA version 11.0 software. The physicochemical properties were predicted using ProtParam at the following online website “https://web.expasy.org/protparam” (accessed on 9 May 2023). SOPMA online software, “http://npsa-pbil.ibcp.fr/cgi-bin” (accessed on 9 May 2023), was used to predict the secondary structure. The tertiary structural was predicted using SWISS-MODEL online software, “https://www.swissmodel.expasy.org” (accessed on 9 May 2023). SOPMA used PHD, GOR, Levin homology prediction, SOPMA, and dual prediction methods to predict the secondary structure of proteins, and synthesizes this into one result. SWISS-MODEL is a homology modelling of protein structures. STRING online software (version 11.5), “https://cn.string-db.org” (accessed on 9 May 2023), was used to analyze the protein interactions.

### 2.11. Statistical Analyses

Data statistical analysis was performed using one-way ANOVA or Student’s *t*-test using IBM SPSS 22.0 software. The data were indicated as mean ± standard error. GraphPad Prism 8.0 was used to draw the pictures. Statistically significant differences were considered at *p* < 0.05. * *p* < 0.05, and ** *p* < 0.01 indicates a significant difference, and ns means no significant difference. A Chi-square test was used to test whether the SV in the *MYL4* gene was in a Hardy–Weinberg balance in Ningxiang pigs and Large White pigs. When *p* > 0.05, it was in a Hardy–Weinberg balance, and when *p* < 0.05, it was in a Hardy–Weinberg imbalance. When polymorphism information content (PIC) is >0.5, the locus is highly polymorphic. When 0.25 < PIC < 0.5, then the locus is moderately polymorphic, and when PIC is <0.25, the locus is considered to be low polymorphic. ImageJ (Version 1.49) software was used for the cell count and to calculate the relative gray value of the protein.

## 3. Results

### 3.1. SV in MYL4 Gene of Ningxiang Pigs and Large White Pigs

**Figure 1:**
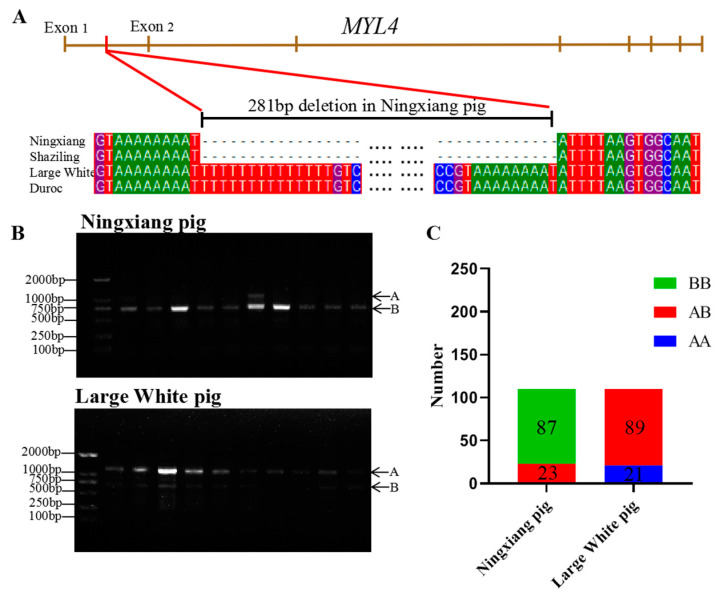
Genotype distribution of the SV of the *MYL4* gene in Ningxiang pigs and Large White pigs. (**A**) 281 bp deletion in the first intronic of *MYL4* of Ningxiang pig. (**B**) The typical agarose gel electrophoresisand analysis result, A represent 1036 bp, B means 755 bp. (**C**) The genotype distribution of the SV in the *MYL4* gene of Ningxiang pigs and Large White pigs.

As shown in Figure 1A, the 281 bp deletion in the first intronic of *MYL4* of Ningxiang pig. The DNA of the Ningxiang pigs (n = 110) and Large White pigs (n = 110) were collected, and then the genotype distributions of the SV in the *MYL4* gene of Ningxiang pigs and Large White pigs were detected using PCR and agarose gel electrophoresis. The production length of primer ssc-*MYL4*-1 is 1036 bp. Here, the genotype distributions of the SV in the *MYL4* gene were defined as follows: the *BB* genotype represents a deletion homozygote (755 bp), the *AB* genotype represents deletion heterozygotes (1036 bp and 755 bp), and the *AA* genotype is a non-deletion homozygote (1036 bp). The typical agarose gel electrophoresis pictures of the genotype distribution of the SV in the *MYL4* gene of Ningxiang pigs and Large White pigs are listed in Figure 1B. As shown in Figure 1B,C, the *AB* genotype (n = 23) and *BB* genotype (n = 87) were detected in Ningxiang pigs, and the *AB* genotype (n = 89) and *AA* genotype (n = 21) were detected in Large White pigs. The detailed detection results of the SVs in the *MYL4* gene in Ningxiang pigs and Large White pigs are shown in Table 3. The dominant genotype was *BB* in Ningxiang pigs (79.09%), but it was *AB* in Large White pigs (80.91%). The frequency of allele A in Ningxiang pigs (10%) is lower than that in Large White pigs (59.55%). In addition, the genotype frequency of Ningxiang pigs was in a Hardy–Weinberg balance (*p* > 0.05), while the genotype frequency of Large White pigs was in a Hardy–Weinberg imbalance (*p* < 0.05). PIC analysis revealed that the PIC of Ningxiang pigs (PIC = 0.17) was lower than that of Large White pigs (PIC = 0.37).

### 3.2. cDNA Cloning and Sequence Analysis of MYL4 in Ningxiang Pigs

**Figure 2:**
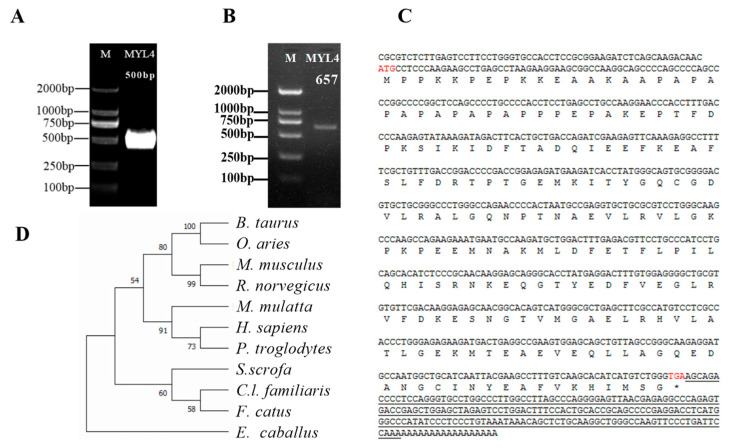
Acquisition of the cDNA sequence of the *MYL4* gene of Ningxiang pigs. (**A**) The 500 bp 3′ RACE products of the *MYL4* gene. (**B**) The 657 bp PCR products of the *MYL4* gene. (**C**) Nucleotide and amino acid sequences of the *MYL4* of Ningxiang pigs, ATG is start codon, TGA is stop codon, underline part is 3′-UTR. (**D**) Phylogenetic tree of MYL4 protein.

**Figure 3:**
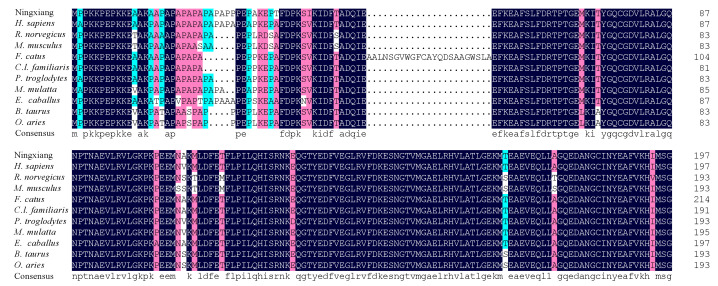
Multiple amino acid sequence alignments of MYL4. Black indicates 100% conserved sequences, pink means the homology is ≥75%, and blue means the homology is ≥50%.

Using 3′ RACE, 500 bp 3′ RACE products of the *MYL4* gene were cloned (Figure 2A). In addition, 657 bp PCR products of the *MYL4* gene were obtained (Figure 2B). Then, two fragments were stitched together, and 857 bp cDNA sequences were obtained. The sequence was consistent with the sequence of the porcine *MYL4* gene in the NCBI database, encoding the same amino acid sequence. As shown in Figure 2C, the *MYL4* cDNA contained a 594 bp ORF encoding a 197 amino acid peptide, the start codon is ATG, and the stop codon is TGA. Phylogenetic trees were constructed using MEGA. As shown in Figure 2D, the MYL4 protein evaluated in this study shared a close evolutionary position with *Canis lupus familiaris* and *Felis catus* MYL4 proteins. The amino acid sequences of MYL4 were aligned with the amino acid sequences of the other 10 species when using DNAMAN. The amino acid sequences used in this study were obtained from GeneBank, and the accession numbers are listed in (Supplementary Table S1). The homology analyses indicated that the MYL4 protein of Ningxiang pigs shared 95.94, 89.34, 87.31, 78.97, 95.43, 92.89, 89.34, 94.42, 92.39, and 92.89% sequence similarity with the MYL4 of *Homo sapiens*, *Rattus norvegicus*, *Mus musculus*, *F. catus*, *C.l. familiaris*, *Pan troglodytes*, *Macaca mulatta*, *Bos taurus*, *Equus caballus*, and *Ovis aries* (Figure 3).

### 3.3. Prediction of the Structures and Features of MYL4 Protein

**Figure 4:**
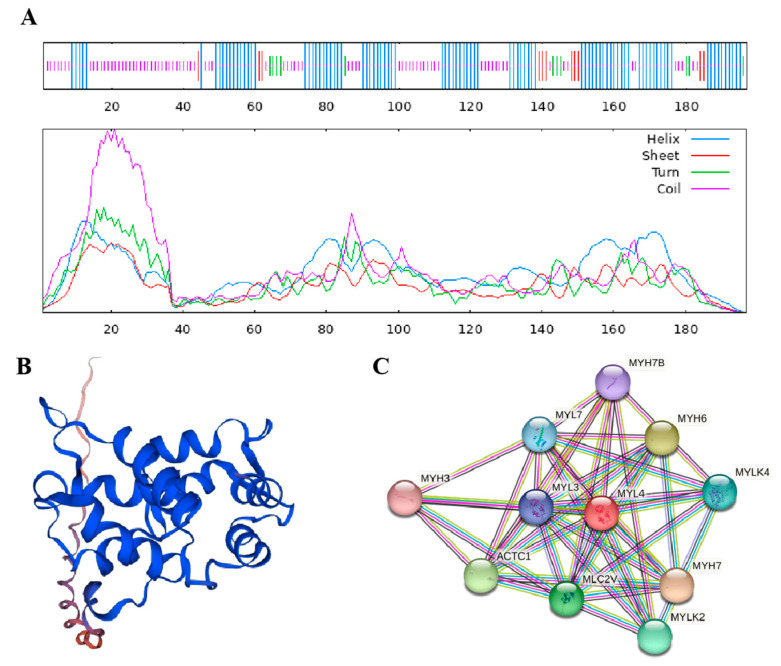
Prediction of the structures and features of MYL4 protein of Ningxiang pigs. (**A**) Predicted secondary structures of the MYL4 protein. (**B**) Predicted tertiary structures of the MYL4 protein. (**C**) Protein interaction analysis of the MYL4 protein.

The physicochemical properties of the MYL4 protein were predicted using ProtParam, and the results are shown in Table 4. The molecular weights were about 22 kDa, and the theoretical pI, aliphatic index, instability, and the grand average of hydropathicity were 4.98, 67.97, 58.38, and −0.535, respectively. Secondary protein structural analysis showed that the MYL4 protein of Ningxiang pigs comprised 46.70% α-helix, 5.58% β-turn, 5.58% extended strand, and 42.13% random coil (Figure 4A), indicating that α-helix and random coils were the dominant structural features of MYL4 proteins. The tertiary structure of the MYL4 protein was predicted using SWISS-MODEL, and the predictions are depicted in Figure 4B, which is consistent with the secondary structure prediction results. As shown in Figure 4C, MYL4 interacted with MYL3, MYL7, MYH3, MYH6, MYH7, MYH7B, ACTC1, MLC2V, MYLK, and MYLK4.

### 3.4. Profiles Analysis of MYL4 Gene Expression in Ningxiang Pigs and Large White Pigs

**Figure 5:**
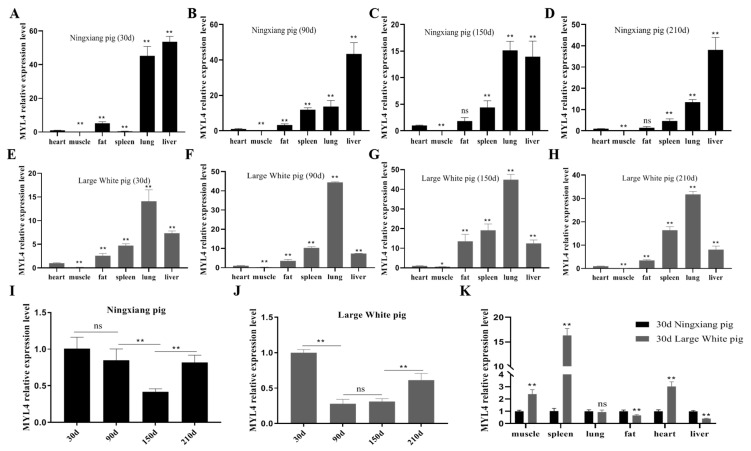
The expressions of *MYL4* in different tissues of Ningxiang pigs and Large White pigs. (**A**–**D**) The expression profiles of *MYL4* in different tissues of 30-, 90-, 150-, and 210-day-old Ningxiang pigs. (**E**–**H**) The expression profiles of *MYL4* in different tissues of 30-, 90-, 150-, and 210-day-old Large White pigs (**I**) The expression levels of *MYL4* in *Longissimus dorsi* muscles of Ningxiang pigs at 30, 90, 150 and 210 days old. (**J**) The expression levels of *MYL4* in *Longissimus dorsi* muscles of Large White pigs at 30, 90, 150 and 210 days old. (**K**) The expression profiles of *MYL4* in 6 tissues of 30 days old Ningxiang pigs and 30 days old Large White pigs. ** *p* < 0.01, ns means no significant difference. n = 3.

RT-qPCR was used to investigate the expression levels of *MYL4* in different tissues and different development periods of Ningxiang pigs and Large White pigs. As shown by the results of RT-qPCR, *MYL4* was widely expressed across tissues in Ningxiang pigs and Large White pigs. *MYL4* is expressed in the lungs of Ningxiang pigs and Large White pigs, with the highest expression, but in the muscles, the lowest expression (Figure 5A–H). The temporal expression profiles of *MYL4* in the muscle showed the highest expression levels 30 days after birth (Figure 5I,J). Further comparing the expression of *MYL4* in different tissues after 30 days for NingXiang pigs and Large White pigs, the results indicated that the expression of *MYL4* in the muscles, spleens, and hearts of Large White pigs was significantly higher than that of Ningxiang pigs (*p* < 0.01), while the expression of *MYL4* in the fat and livers of Large White pigs was significantly lower than that of Ningxiang pigs (*p* < 0.01) (Figure 5K).

### 3.5. Knockdown and Overexpression of MYL4

**Figure 6:**
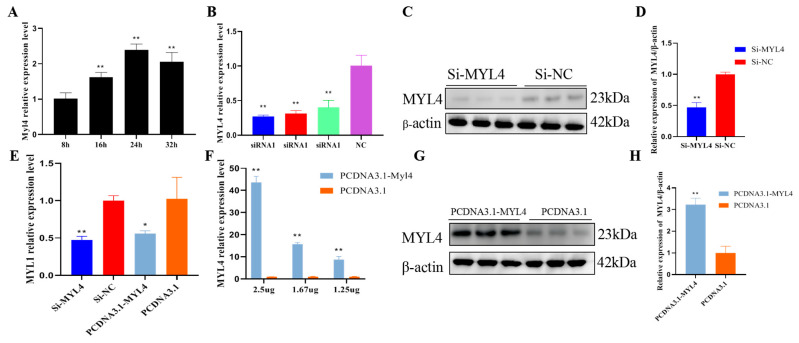
*MYL4* was successfully inhibited or overexpressed in C2C12 cells. (**A**) Relative expression levels of *MYL4* in C2C12 cells after proliferation for 8 h, 16 h, 24 h, and 32 h. (**B**) Relative expression levels of *MYL4* were detected using RT-qPCR 24 h after transfection with Si-*MYL4*, and Si-NC. (**C,D**) The protein expression of MYL4 was detected using Western blot in C2C12 cells 48 h after transfection with Si-*MYL4* and Si-NC. (**E**) Relative expression levels of *MYL1* were detected using RT-qPCRafter transfection with Si-*MYL4*, Si-NC, PCDNA3.1-*MYL4* and PCDNA3.1. (**F**–**H**) Relative expression levels of *MYL4* were detected using RT-qPCR, and the protein expression was detected by Western blot after transfection with PCDNA3.1-*MYL4* and PCDNA3.1. * *p* < 0.05 and ** *p* < 0.01.

The endogenous *MYL4* in proliferating C2C12 cells was detected through the use of RT-qPCR, and the cells were collected at 8 h (8 h after the cells were seeded), 16 h, 24 h, and 32 h, respectively. From the results of the RT-qPCR, *MYL4* was the most strongly expressed at 24 h, which then gradually downregulated (Figure 6A). *MYL4* was successfully inhibited or overexpressed in C2C12 cells by transfecting the si-*MYL4* or *MYL4*-plasmid. As shown in Figure 6B–D, *MYL4* was successfully inhibited, and siRNA1 had the highest inhibition efficiency (*p* < 0.01). The transfection of 2.5 ug of PCDNA3.1-*MYL4* had the highest overexpression efficiency (*p* < 0.01) (Figure 6F–H). Next, the mRNA expressions of *MYL1*, *MYL2*, and *MYL3* were detected by means of RT-qPCR in C2C12 cells proliferating for 24 h, and it was found that *MYL1* mRNA was expressed in C2C12 cells, while *MYL2* and *MYL3* were not detected. RT-qPCR was used to explore the effect of *MYL4* on other light chain genes after the knockdown and overexpression of *MYL4*. As shown in the RT-qPCR results, the mRNA expression of *MYL1* insignificantly decreased when *MYL4* was inhibited (*p* < 0.01), and the mRNA expression of *MYL1* also insignificantly decreased when *MYL4* was overexpressed (Figure 6E) (*p* < 0.05).

### 3.6. MYL4 Inhibits C2C12 Cell Proliferation

**Figure 7:**
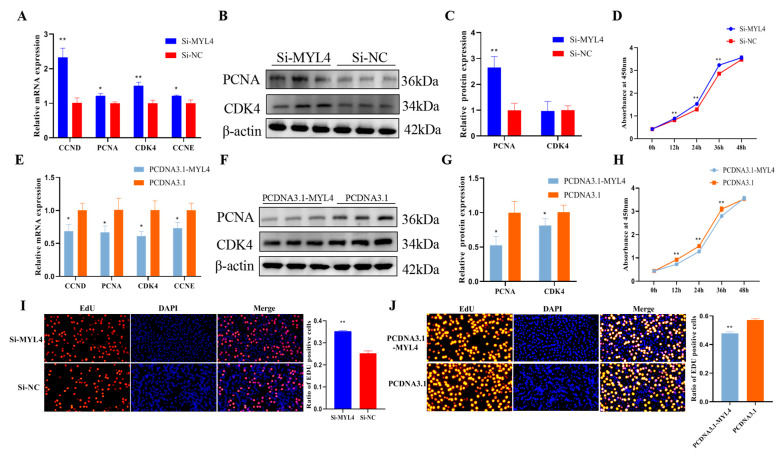
*MYL4* inhibits C2C12 cell proliferation. (**A**–**C**) Relative expression levels of *CCND*, *PCNA*, *CDK4*, and *CCNE* were detected through the use of RT-qPCR, and the protein expressions of PCNA, CDK4 were detected using Western blot after transfection with Si-*MYL4* and Si-NC. (**D**) Cell viability was measured using a CCK8 kit after transfection with Si-*MYL4* and Si-NC. (**E**–**G**) Relative expression levels of *CCND*, *PCNA*, *CDK4*, and *CCNE* were detected through the use of RT-qPCR, and the protein expressions of PCNA and CDK4 were detected using Western blot after transfection with PCDNA3.1-*MYL4* and PCDNA3.1. (**H**) Cell viability was measured using a CCK8 kit after transfection with PCDNA3.1-*MYL4* and PCDNA3.1. (**I**,**J**) Cell proliferation was measured using an EdU assay after transfection with Si-*MYL4*/Si-NC, PCDNA3.1-*MYL4* and PCDNA3.1. * *p* < 0.05 and ** *p* < 0.01. n = 3.

Concerning the effect of *MYL4* on C2C12 cell proliferation, and as shown by the result of RT-qPCR and Western blot, the knockdown of *MYL4* in C2C12 cells caused a marked increase in the mRNA expression of *CCND*, *PCNA*, *CDK4*, and *CCNE* (*p* < 0.05) and the protein levels of PCNA (*p* < 0.01) (Figure 7A–C). The results of the CCK8 analysis indicated that the knockdown of *MYL4* dramatically promoted C2C12 cell proliferation (*p* < 0.01) (Figure 7D). The overexpression of *MYL4* showed that the mRNA expression of *CCND*, *PCNA*, *CDK4* and *CCNE* significantly decreased (Figure 6E) (*p* < 0.05), and the protein of PCNA and CDK4 significantly decreased (Figure 6F,G) (*p* < 0.05). The CCK8 analysis results also indicated that an overexpression of *MYL4* could inhibit C2C12 cells proliferation (Figure 7H) (*p* < 0.01). EdU staining was used to further confirm the effect of *MYL4* on cell proliferation, and the results indicated that *MYL4* knockdown significantly promoted C2C12 cell proliferation (*p* < 0.01) (Figure 7I), and the overexpression of *MYL4* markedly inhibited C2C12 cell proliferation (*p* < 0.01) (Figure 7J). In general, these results suggested that *MYL4* could inhibit C2C12 cell proliferation.

### 3.7. MYL4 Promotes C2C12 Cells Apoptosis

**Figure 8:**
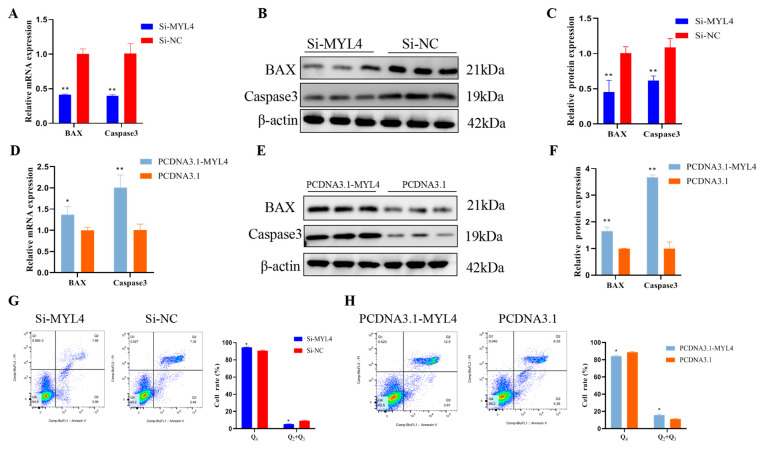
*MYL4* promotes C2C12 cell apoptosis. (**A**) The mRNA expression of *BAX* and *Caspase3* were detected through the use of RT-qPCR 24 h after transfection with Si-*MYL4* and Si-NC. (**B**,**C**) The protein expressions of BAX and Caspase3 in C2C12 cells were detected using Western blot after transfection with Si-*MYL4* and Si-NC. (**D**) Relative expression levels of *BAX* and *Caspase3* were detected through the use of RT-qPCR after transfection with PCDNA3.1-*MYL4* and PCDNA3.1. (**E**,**F**) The protein expressions of BAX and Caspase3 in C2C12 cells were detected via Western blot after transfection with Si-*MYL4* and Si-NC. (**G**,**H**) The rate of C2C12 cell apoptosis was determined through the use of flow cytometry after transfection with Si-*MYL4*/Si-NC, PCDNA3.1-*MYL4*/PCDNA3.1. * *p* < 0.05 and ** *p* < 0.01. n = 3.

To explore the effect of *MYL4* on C2C12 cell apoptosis, Si-*MYL4*, Si-NC, PCDNA3.1-*MYL4*, and PCDNA3.1 were transfected into C2C12 cells. As shown in Figure 8A, the mRNA expression of *BAX* and *Caspase3* significantly decreased after transfection with Si-*MYL4* (*p* < 0.01). The Western blot results showed that the protein expression of BAX and Caspase3 significantly decreased in Si-*MYL4* when compared with Si-NC (*p* < 0.01) (Figure 8B,C). The overexpression of *MYL4* showed an opposite result (Figure 8D–F). This result indicated that *MYL4* promotes C2C12 cell apoptosis. To further confirm the effect of *MYL4* on cell apoptosis, cell apoptosis was detected using flow cytometry. The flow cytometry results showed that the number of apoptosis cells (Q2 + Q3) in the Si-*MYL4* group was significantly lower than that in Si-NC (*p* < 0.05) (Figure 8G), and the number of apoptosis cells in the PCDNA3.1-*MYL4* group was much higher than that in the PCDNA3.1 group (*p* < 0.05) (Figure 8H). In general, the above results suggested that *MYL4* could promote C2C12 cell apoptosis.

### 3.8. Expression Pattern of MYL4 during the Differentiation of C2C12 Cells

**Figure 9:**
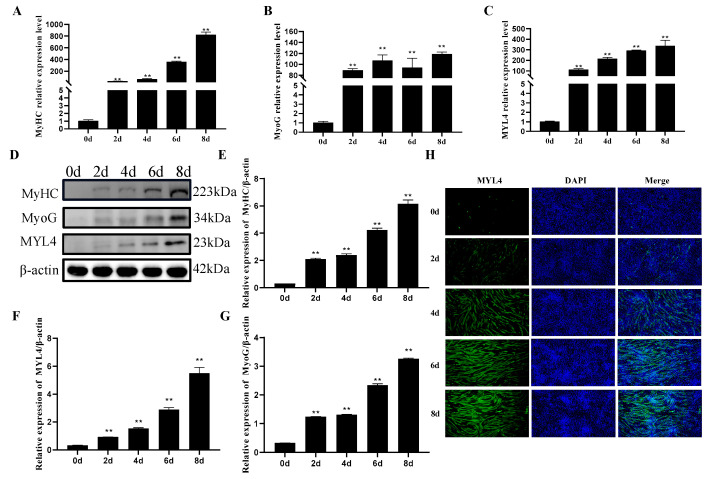
The expression patterns of *MyHC*, *MyoG*, and *MYL4* in C2C12 cell differentiation for 0, 2, 4, 6, and 8 days. (**A**–**C**) Relative expression levels of *MyHC*, *MyoG*, and *MYL4* in C2C12 cell differentiation for 0–8 d, as detected through the use of RT-qPCR. (**D**) Change in MyHC, MyoG, and MYL4 expression levels in C2C12 cell differentiation for 0–8 d, as detected via Western blot. (**E**–**G**) Gray scanning of MyHC, MyoG, and MYL4 is shown in Figure 8D. (**H**) MYL4 protein expression in C2C12 cell differentiation for 0–8 d, as detected via immunofluorescence. ** *p* < 0.01. n = 3.

To detect the expression levels of *MYL4* at different stages of C2C12 cell differentiation, C2C12 cells were collected at differentiation periods of 0 d, 2 d, 4 d, 6 d, and 8 d, respectively. RT-qPCR and Western blot were used to detect the mRNA expressions and protein expressions of *MyHC, MyoG*, and *MYL4*. As shown in Figure 9A,B, the mRNA expression levels of *MyHC* and *MyoG* were both gradually upregulated as C2C12 cell differentiation progressed. In addition, the result of the Western blot indicated that the expression levels of *MyHC* and *MyoG* increased significantly during C2C12 cell differentiation (Figure 9D–F). These results demonstrated that the C2C12 cells were well differentiated. As shown in Figure 9C,G, the mRNA and protein expression levels of *MYL4* increased during C2C12 cell differentiation. The fluorescence intensity of MYL4 was enhanced with the extension of the differentiation time, as shown through immunofluorescence staining (Figure 9H). These results indicated that *MYL4* may play an important role in C2C12 cell differentiation.

### 3.9. MYL4 Promotes C2C12 Cells Differentiation

**Figure 10:**
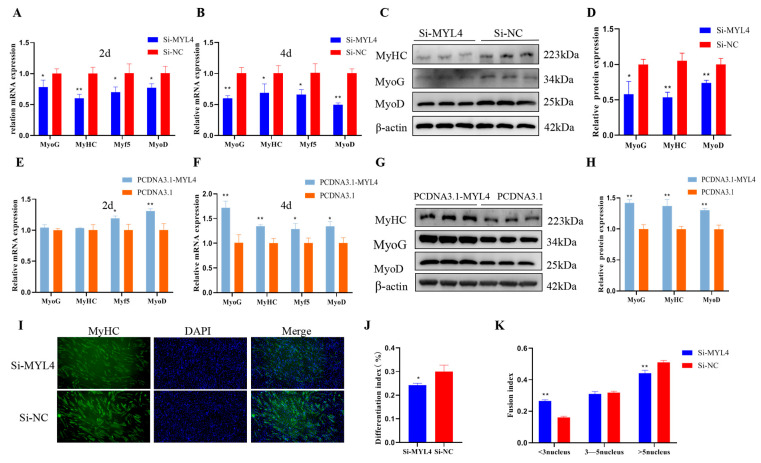
*MYL4* promotes C2C12 cell differentiation. (**A**,**B**) Relative expression levels of *MyoG*, *MyHC*, *Myf5*, and *MyoD* were detected via RT-qPCR at the 2nd and 4th days of myoblast differentiation after transfection with Si-*MYL4* and Si-NC. (**C**,**D**) The protein expression levels of MyHC, MyoG, and MyoD were detected via Western blot at the 4th day of differentiation after transfection with Si-*MYL4* and Si-NC. (**E**,**F**) Relative expression levels of *MyoG*, *MyHC*, *Myf5*, and *MyoD* were detected through the use of RT-qPCR at the 2nd and 4th days of myoblast differentiation after transfection with PCDNA3.1-*MYL4* and PCDNA3.1. (**G**,**H**) The protein expression levels of MyHC, MyoG, and MyoD were detected via Western blot at the 4th day of differentiation after transfection with PCDNA3.1-*MYL4* and PCDNA3.1. (**I**) Immunofluorescence analysis of MyHC in C2C12 myoblasts at the 4th day of differentiation after transfection with Si-*MYL4* and Si-NC. (**J**,**K**) The differentiation index and fusion index of myoblasts. * *p* < 0.05 and ** *p* < 0.01. n = 3.

To explore the effect of *MYL4* on C2C12 cell differentiation, Si-*MYL4*, Si-NC, PCDNA3.1-*MYL4*, and PCDNA3.1 were transfected into C2C12 cells. The cells were induced to differentiate for 2 d and 4 d. As shown in the results of RT-qPCR and Western blot, the mRNA expression levels of *MyoG*, *MyHC*, *Myf5*, and *MyoD* significantly decreased (*p* < 0.05), and the protein expression levels of MyoG, MyHC, and MyoD significantly decreased after the knockdown of *MYL4* (*p* < 0.05) (Figure 10A–D). The overexpression of *MYL4* could upregulate the mRNA expression of *MyoG*, *MyHC*, *Myf5*, and *MyoD* (*p* < 0.05)*,* and the protein expression levels of MyoG, MyHC, and MyoD (*p* < 0.01) (Figure 10E–H). These results indicated that *MYL4* was a positive regulator of C2C12 cell differentiation. Then, immunofluorescence was used to further investigate the effect of *MYL4* on C2C12 cell differentiation (Figure 10I), and the results indicated that the differentiation index decreased from 30.01% to 24.25% (*p* < 0.05) (Figure 10J). Fewer than three nuclear myotubes in the Si-*MYL4* group was significantly higher than that in Si-NC (*p* < 0.01), and more than five nuclear myotubes in the Si-*MYL4* group was significantly lower than that in Si-NC (*p* < 0.01) (Figure 10J,K). The above results indicate that *MYL4* could promote C2C12 cell differentiation.

## 4. Discussion

SVs are large genomic alterations. It has been reported that large genomic SVs have a greater effect on gene expression than single-nucleotide variations and that SVs have a greater effect on gene expression and function [19]. Ma et al. proved that the deletion of 281 bp in the first intron of the *MYL4* gene can promote fat deposition in Ningxiang pigs; thus, does the deletion of 281 bp in the first intron of the *MYL4* gene affects the development of skeletal muscle in Ningxiang pigs? In this study, genotype *BB* was found to exist mainly in Ningxiang pigs, and genotype *AB* existed mainly in Large White pigs, and the frequency of A allele in Ningxiang pigs was lower than that in Large White pigs. In addition, the genotype frequency of Ningxiang pigs was in a state of Hardy–Weinberg balance, but the genotype frequency of Large White pigs was in a state of Hardy–Weinberg imbalance, which may be associated with population loss or the degree of inbreeding. The PIC values of the SV in the *MYL4* gene of Ningxiang pigs is 0.17, and those results indicate that the variation in the SV in the *MYL4* gene of Ningxiang pigs is stable. In recent decades, more and more full-length sequences of genes have been obtained through the use of the RACE method [20]. Wu et al. cloned the cDNA sequence of porcine *MYL4* for the first time, which provided a basis for revealing the structure and function of the porcine *MYL4* gene [21]. This research showed that the amino acid sequence of the MYL4 in Ningxiang pigs was consistent with the amino acid sequence of the porcine MYL4 in the NCBI database, and the MYL4 protein of Ningxiang pigs shared 87.31% sequence similarity with *M. musculus*. 3′-UTR of mRNA is not only the hub of post-transcriptional control but also the target of binding to miRNA. It may specifically cleave mRNA when the 3′-UTR sequence of the mRNA is completely complementary to that of the miRNA [22]. This research cloned the cDNA sequence of the *MYL4* gene of Ningxiang pigs, obtained the 3′-UTR sequence, and predicted its physicochemical properties. It provides a basis for further obtaining the full-length sequence of *MYL4* in Ningxiang pigs, finding its combined miRNA, and exploring its regulatory mechanism. *MYL4* is ubiquitously present in fetal and neonatal cardiac muscle and is also involved in muscle development and growth in pigs [23,24]. This research found that *MYL4* was widely expressed in the tissues of Ningxiang pigs and Large White pigs and highly expressed in the liver and lung, but it is rarely expressed in the *Longissimus dorsi*. The results of this study are similar to other *MYL4*-related studies [25,26,27]. Ningxiang pigs are fatty-type pigs with a high IMF content, while Large White pigs are lean-type pigs with a high lean meat content [28,29]. SV can influence the gene dosage directly or indirectly through different mechanisms, thereby causing phenotypic variation and even disease in livestock and poultry [30]. In this research, the expression of *MYL4* in the muscle of 30-d Ningxiang pigs was lower than that of 30-d Large White pigs; therefore, we speculate that the deletion of 281 bp in the first intron of the *MYL4* gene in Ningxiang pigs potentially drives a decrease in the transcription of *MYL4* in the muscle of Ningxiang pigs. Our results are similar to the results of Zhou [31]. In addition, there may be genetic differences other than the SV between the two pigs that cause the expression differences in *MYL4*.

Given that C2C12 cells are the classical model used in the study of skeletal muscle growth and development, this study explored the role of *MYL4* in skeletal muscle development in C2C12 cells [32]. Studies have shown that *MYL4* begins to be expressed at the beginning of myoblast differentiation, and other studies have shown that *MYL4* is expressed prior to the shaping of the myotubes [33,34]. This research showed that a high level of *MYL4* gene mRNA expression was detected at 8 h after the proliferation of C2C12 cells, and MYL4 protein expression was also detected at 48 h after proliferation, which is consistent with the results of Zhan [35]. The mRNA expression of *MYL1* was detected at 8 h after the proliferation of C2C12 cells, but *MYL2* and *MYL3* were not detected in C2C12 cells. Previous studies reported that *MYL2* and *MYL3* were expressed only in mature muscles [9,36,37], and our results were consistent with the existing literature. Myoblast proliferation is the key step to muscle regeneration. *PCNA*, *CDK4*, *CCND*, and *CCNE* are the key genes in cell proliferation; the expression of these genes reflects cell proliferation [38,39]. In this research, the function of *MYL4* in C2C12 cells was determined using overexpression and knockdown experiments. The results showed that the knockdown of *MYL4* could promote C2C12 cell proliferation, and the overexpression of *MYL4* showed the opposite result. This research indicated that *MYL4* could promote C2C12 cell apoptosis. *MyoD*, *MyoG*, *Myf5*, and *MyHC* are muscle differentiation marker genes [40,41]. Our results showed that the mRNA and protein expression levels of *MyoG* and *MyHC* increased upon myogenic stimuli, indicating that the C2C12 cells were well-differentiated. Subsequently, the mRNA and protein expression levels of *MYL4* were found to be rarely expressed on the 0th day and increased upon myogenic stimuli. Given that *MYL4* increased during myogenic differentiation, we further explored the effect of *MYL4* on C2C12 cell differentiation. In this research, the knockdown of *MYL4* could inhibit C2C12 cell differentiation, and the overexpression of *MYL4* promotes C2C12 cell differentiation. These results indicated that *MYL4* is a positive regulator of skeletal muscle differentiation. In summary, the above results indicate that *MYL4* inhibits C2C12 proliferation and promotes C2C12 cell apoptosis and differentiation.

## 5. Conclusions

In this study, we verified the presence of SV in the *MYL4* gene. The genotype distribution of SV in *MYL4* was detected in Ningxiang pigs and Large White pigs. The main genotypes of Ningxiang pigs and Large White pigs were *BB* and *AB*, respectively. The cDNA sequence of *MYL4* was cloned, and the physicochemical properties of *MYL4* were analyzed. *MYL4* has been demonstrated to inhibit proliferation and promote apoptosis and differentiation in myoblasts. These results laid a foundation for the molecular mechanism of pork quality improvement and muscle development.

## Figures and Tables

**Table 1:** Sequences of siRNA.

siRNA	Forward (5′→3′)	Reverse (5′→3′)
siRNA1	GCUGACCAGAUCGAAGAAUTT	AUUCUUCGAUCUGGUCAGCTT
siRNA2	GCUGCGGGUCUUUGACAAATT	UUUGUCAAACACCCGCAGCTT
siRNA3	GCAUCAACUAUGAAGCCUUTT	AAGGCUUCAUAGUUGAUGCTT

**Table 2:** Primers for RT-qPCR.

Gene	Primer Sequence (5′→3′)	Tm (°C)	Length (bp)
*ssc-MYL4*	F: CAGCCCAGTCTCCCATCT	60	375
	R: GCAGCACCTCGGCATTAG		
*mmu-MYL4*	F: GAAACCCGAGCCTAAGAA	60	175
	R: AGTCCGGTCAAACAATGAA		
*CCNE*	F: CCTCTGCTCGGGTGTTGTAG	60	72
	R: TCTGCATCCCACACTTGCTC		
*CCND*	F: TCAAGTGTGACCCGGACTG	60	235
	R: GCTCCTTCCTCTTTGCGGG		
*PCNA*	F: GCCGAGACCTTAGCCACATT	60	229
	R: GTAGGAGACAGTGGAGTGGC		
*CDK4*	F: CGAGCGTAAGGCTGATGGAT	60	177
	R: CCAGGCCGCTTAGAAACTGA		
*Caspase3*	F: GCTTGGAACGGTACGCTAAG	60	112
	R: CCACTGACTTGCTCCCATGT		
*BAX*	F: CCAGGATGCGTCCACCAA	60	196
	R: AAAGTAGAAGAGGGCAACCAC		
*MyoG*	F: CAATGCACTGGAGTTCGGT	60	134
	R: CTGGGAAGGCAACAGACAT		
*MyHC*	F: CGGTCGAAGTTGCATCCCT	60	141
	R: GAGCCTCGATTCGCTCCTTT		
*Myf5*	F: CAGGAATGCCATCCGCTACA	60	78
	R: CCCGGCAGGCTGTAATAGTT		
*MyoD*	F: AAGACGACTCTCACGGCTTG	60	169
	R: GCAGGTCTGGTGAGTCGAAA		
*Gapdh*	F: AGGGCATCCTGGGCTACACT	60	166
	R: TCCACCACCCTGTTGCTGTAG		
*MYL1*	F: GGGAACCCCAGCAATGAAGA	60	132
	R: GAAGACACGCAGACCCTCAA		
*MYL2*	F: CTGACGTCACCGGCAATCTT	60	195
	R: GGCAACTCCCATCTTCTCCT		
*MYL3*	F: GCCAAGCATCTCCCAACCAT	60	115
	R: GGGCCAGGAAAGACTACCAC		

**Table 3:** The analysis of the genetic diversity of *MYL4* in Ningxiang pigs and Large white pigs.

Breeds	Genotype Frequency (%)			Gene Frequency (%)		χ^2^	*p*	PIC
	AA	AB	BB	A	B			
Ningxiang	0	20.91	79.09	10	90	1.50	>0.05	0.17
Large White	19.09	80.91	0	59.55	40.45	50.77	<0.05	0.37

**Table 4:** Physicochemical properties of MYL4 protein.

Physicochemical Properties	MYL4
Amino acid	197
Molecular weight (Da)	21,606.64
Theoretical pI	4.98
Number of atoms	3031
Asp + Glu	32
Arg + Lys	23
Extinction coefficients	4595
Aliphatic index	67.97
Instability index	58.38
Grand average of hydropathicity	−0.535

## Data Availability

The data analyzed during the current study are available from the corresponding author on reasonable request.

## Supplementary Materials

The following supporting information can be downloaded at: https://www.mdpi.com/article/10.3390/genes14061267/s1, Table S1: The accession numbers of *MYL4* in different species.
